# Perceived Family Functioning in Relation to Energy Intake in Adolescent Girls with Loss of Control Eating

**DOI:** 10.3390/nu10121869

**Published:** 2018-12-02

**Authors:** Manuela Jaramillo, Natasha L. Burke, Lauren B. Shomaker, Sheila M. Brady, Merel Kozlosky, Jack A. Yanovski, Marian Tanofsky-Kraff

**Affiliations:** 1 Section on Growth and Obesity, *Eunice Kennedy Shriver* National Institute of Child Health and Human Development (NICHD), National Institutes of Health (NIH), DHHS, 10 Center Drive, Bethesda, MD 20892-1103, USA; Manuela.Jaramillo@nih.gov (M.J.); BradyS@mail.nih.gov (S.M.B.); jy15i@nih.gov (J.A.Y.); 2 Department of Medical and Clinical Psychology, Uniformed Services University of the Health Sciences (USUHS), 4301 Jones Bridge Road, Bethesda, MD 20814, USA; 3 Department of Psychology, Fordham University, 411 East Fordham Road, Bronx, NY 10458, USA; NBurke12@fordham.edu; 4 Department of Human Development and Family Studies, Colorado State University, 303 Behavioral Sciences Building, 1570 Campus Delivery, Fort Collins, CO 80523, USA, Lauren.Shomaker@colostate.edu; 5 Nutrition Department, Clinical Center, NIH, DHHS, 10 Center Drive, Bethesda, MD 20892, USA; KozloskyM@cc.nih.gov

**Keywords:** loss of control eating, obesity, BMI, adolescent, females, family functioning, energy intake

## Abstract

Family functioning is hypothesized to influence the development, maintenance, and treatment of obesity and eating disorders. However, there are limited data examining family functioning in relation to energy intake in the laboratory among youth at high-risk for eating disorders and excess weight gain. Therefore, we examined the relationship between perceived family functioning and energy intake during a laboratory test meal designed to model a binge episode. We performed hierarchical multiple regression analyses among 108 adolescent girls in an excess weight gain prevention trial. Participants were at high-risk for eating disorders and excess weight gain due to reports of loss of control eating (LOC) and high body mass index (BMI). Participants completed the Family Adaptability and Cohesion Scale III to assess family adaptability and cohesion. Following an overnight fast, girls consumed lunch from a laboratory test meal. Poorer family adaptability, but not cohesion, was associated with lower percentage of total energy intake from protein and greater percentage of total energy intake from carbohydrates. Neither adaptability nor cohesion were significantly associated with total intake. We conclude that among girls with LOC eating and high BMI, poor reported family adaptability is associated with greater consumption of obesity-promoting macronutrients during binge episodes. Directionality and temporality of this association between unhealthy consumption and family rigidity requires further study.

## 1. Introduction

Given the high prevalence of obesity among adolescents [1] and the relatively few effective treatment options for this age group [2], elucidating psychological factors that promote excess weight gain is warranted for the development of novel approaches. Loss of control eating (LOC), the subjective feeling that one cannot stop or control what or how much one consumes [3], may be one such factor. With approximately 33% of children with overweight or obesity engaging in LOC eating [4], LOC eating has been shown to be a robust predictor of excess weight and fat gain [5,6,7], exacerbate some components of the metabolic syndrome [8], and predict the development of partial or full-syndrome binge eating disorder [9,10]. Data have shown that, in general (for example, outside of the laboratory environment), youth with LOC eating consume more highly palatable, energy dense foods than youth without LOC eating [11,12,13], which may provide a mechanism for observed prospective outcomes.

The interpersonal model of binge eating disorder proposes that the link between difficulties in social functioning and LOC eating is largely mediated by negative affect resulting from relationship problems [14]. This model has been applied to adolescent girls with LOC eating [15]. While the theory focuses on any type of salient relationship, data suggest that among adolescent girls with LOC eating, family members may be most relevant to the interpersonal model [16]. Indeed, family dysfunction has been suggested to play a crucial role in child and adolescent overweight and obesity [17,18,19] and disordered eating [20,21,22].

Prior studies have operationalized family functioning as a person’s perceived level of adaptability (i.e., ability of a family system to change its power structure, role relationships, and relationship rules in response to situational and developmental stress) and cohesion (i.e., emotional bonding that family members have toward one another) within their own family [23]. Research has shown that higher and/or balanced levels of perceived adaptability and/or cohesion can both relate to and predict healthy eating behaviors. Generally, it has been found that girls with diagnosed eating disorders (compared to girls without diagnosed eating disorders) report less functional levels of adaptability and/or cohesion [21,24]. For example, studies have shown that adolescents with binge-eating disorder perceive lower familial adaptability than adolescents without binge-eating disorder [25], report higher cohesion and flexibility compared to adolescent girls with anorexia nervosa or bulimia nervosa [26], and report more negative perceived maternal criticism than a control group [27]. Among non-clinical samples of adolescents with overweight, higher reports of overeating relate to lower perceived familial cohesion and adaptability [28], and mothers of adolescents with overweight also report higher psychopathology than mothers of adolescents without overweight [29].

Perceived family functioning has also been shown to relate to energy intake patterns [30,31]. For example, a cross-sectional study of youth and adults with diabetes assessing the relationships among family meal frequency, perceived familial cohesion, and reported intake found that cohesion mediated the relationship between family meal frequency and individuals’ intake as measured through a food frequency questionnaire [32]. Specifically, higher levels of perceived familial cohesion were linked to a greater reported intake of several food items considered healthful, whereas lower levels of perceived familial cohesion were associated with a greater consumption of palatable and high-caloric density items like fried eggs, burgers, and chips. Similar findings were observed in a longitudinal study assessing the relationship between family cohesion and self-reported food intake in adolescents using a food diary. Perceived cohesion was positively related to healthier reported eating behaviors, such as breakfast consumption, and was negatively related to soda intake [31].

One limitation to the existing literature is the use of self-reported intake, which can be under (or over) reported [33,34], particularly among individuals with overweight and obesity [35,36] or those reporting LOC eating [37]. However, no study has examined total or macronutrient intake in relation to family functioning during actual meals, which may be more informative for understanding, and potentially preventing, obesity development. Lastly, there are no data specifically examining the family functioning and test meal intake of adolescent girls with LOC at high-risk for excess weight gain and eating disorders. We therefore related objectively measured intake at a meal designed to model a binge episode to reported familial functioning among high-risk adolescent girls. Given data showing that youth reporting LOC eating episodes tend to consume more energy from carbohydrate and fat and less energy from protein compared to youth not reporting LOC eating episodes [11,38], girls with overweight have greater overall energy consumption [13,39] and youth with LOC tend to have greater liking of foods in a test meal [40]. We hypothesized that lower levels of adaptability and cohesion would relate to greater total energy consumption, a greater percentage of energy consumed from fat and carbohydrates, and a lower percentage of energy consumed from protein.

## 2. Method

### 2.1. Participants

108 adolescent girls aged 12–17 years old and at high-risk for eating disorders and excess weight gain participated in a clinical study for the prevention of LOC eating (ClinicalTrials.gov ID#: NCT00680979). All data for the current analyses were collected at baseline prior to the initiation of any treatment. As previously described [41], inclusion criteria involved being between the 75th and 97th percentile for body mass index (BMI; kg/m^2^) and having undergone at least one LOC episode in the last month. Exclusion criteria included any major medical or psychiatric diagnoses (other than binge eating disorder), regular use of medications that affect weight or appetite, current involvement in psychotherapy or a weight loss program, or weight loss in last 2 months exceeding >3% of body weight. A total of 185 participants were screened of whom 117 were eligible for the study. 108 participants completed the necessary measures for the current analysis. Signed informed consent and assent were obtained from parents and adolescents respectively. This protocol was approved by the Institutional Review Boards at the Eunice Kennedy Shriver National Institute of Child Health and Human Development and the Uniformed Services University of the Health Sciences.

### 2.2. Procedure

Demographic variables (e.g., age and race/ethnicity) were assessed by parent report and questionnaires and interviews were assessed by adolescent report. BMI, adiposity, and energy intake were assessed at the National Institutes of Health Clinical Research Center following an overnight fast initiated the night before at 10 p.m.

BMI and adiposity: BMI was calculated from height and weight. Weight was measured to the nearest tenth of a kilogram with a calibrated digital scale (Scale-Tronix, Wheaton, IL, USA). Height was measured in triplicate to the nearest millimeter with a calibrated electronic stadiometer (Holtain Ltd., Crymych, Wales, UK) and the average of the three heights was used. Fat mass (kg) was measured by dual energy X-ray absorptiometry (DXA) using a Hologic QDR-4500A or Discovery instrument (Bedford, MA, USA) as previously described [41].

LOC eating: The presence of LOC eating over the past month was assessed by the overeating section of the Eating Disorder Examination, Version 14 OD/C.2 (EDE; [42]). The Eating Disorder Examination is a semi-structured diagnostic clinical interview used to assess disordered attitudes and behaviors related to eating, body shape, and weight. This instrument has been shown to be reliable and valid in pediatric samples [43,44].

Family functioning: Family functioning was defined as an adolescent’s perceived level of adaptability and cohesion within their own family [23]. Perceived familial adaptability and cohesion were assessed with the Family Adaptability and Cohesion Scale III (FACES III), a 20-item self-report instrument that measures perceived levels of familial adaptability (e.g., “Parent(s) and children discuss punishment together”) and cohesion (e.g., “Family members like to spend free time with each other”). The FACES has been used frequently in the literature to assess family function and is based on the hypothesis that functional family systems have more balanced levels of adaptability and cohesion than do dysfunctional family systems. Items were rated on a 5-point Likert scale (i.e., almost never, once in a while, sometimes, frequently, almost always) and scales were derived by summing items. The possible subscale score ranges for adaptability and cohesion were 10–50, with higher scores indicating greater (more positive) adaptability or cohesion. The FACES III has exhibited good test–retest reliability for both subscales (adaptability Cronbach’s alpha = 0.80 at the 5-week interval; cohesion Cronbach’s alpha = 0.83 at the 5-week interval) and strong discriminant validity given a low correlation between adaptability and cohesion (*r* = 0.03) [23]. Cronbach’s alpha for the current study was 0.75 for adaptability and 0.87 for cohesion. Considering that the data for the current study was collected between 2008 and 2011, the FACES version used is not the most current as of 2011. However, if adaptability and cohesion are analyzed as continuous variables (as was done in the current study) and not categorical variables, the FACES III is a valid and interpretable measure [23].

Energy intake: At 11:00 a.m., participants were presented with a 9835 kcal buffet test meal with individual items representing a wide assortment of foods that varied in macronutrient composition (12% protein, 51% carbohydrate, and 37% fat across all foods). Adolescents entered the room containing the buffet and were provided with the tape-recorded instruction stating, ‘‘let yourself go and eat as much as you want.” Participants were then left alone in the room until they indicated that they were finished eating. Total consumption of each food item was assessed by weighing the food items before and after presentation to each participant. This paradigm has effectively distinguished adolescents with LOC eating from those without reported LOC eating [11]. Some of the test meal data collected at baseline have been published in previous reports [45,46,47,48].

Pre-meal hunger: A single item-assessment was used to measure pre-meal hunger. The item asked, “how hungry do you feel right now?” and individuals responded on a 1 (Not at all) to 100 (Extremely) sliding scale.

Depressive symptoms: A single item-assessment was used to measure pre-meal depressive symptoms from the Brunel Mood Scale [49]. The item asked participants to select their current level of depression on a 1 (Not at all) to 5 (Extremely) Likert scale.

Post-meal subjective evaluation of LOC eating: A single item-assessment was used to measure post-meal subjective evaluation of LOC eating. The item asked, “how much is the eating you just completed like a meal when you feel ‘loss of control’?” on a 1 (Extremely) to 5 (Not at all) Likert scale.

### 2.3. Statistical Analyses

Analyses were performed using SPSS version 25. Hierarchical multiple regression analyses were conducted with perceived adaptability and cohesion as independent variables and macronutrients (protein, carbohydrates, and fat as a percentage of total energy consumption) and total energy intake as dependent variables. R squared values indicate the proportion of variance in the dependent variable that is explained by the model. Height, body fat (%), lean body mass (kg), age, race, depressive symptoms, pre-meal hunger, and total energy intake (kcal) were considered as covariates in the models predicting percentage of macronutrients. Age, race, depressive symptoms, and pre-meal hunger did not contribute to any model and were removed from analyses. Height was highly correlated with lean body mass (*r* = 0.77) and was not included in analyses to meet multicollinearity assumptions. Macronutrient intake percentages were arcsine square root transformed to achieve normality. All other assumptions for model testing were met. Significance was set at α = 0.05, two-tailed. We also conducted a series of follow up analyses for participants with overweight or obesity (BMI percentile ≥ 85).

## 3. Results

Participants were 108 adolescent girls (M ± SD 14.49 ± 1.66 years) who had above-average weight with a mean BMIz of 1.54 (SD = 0.33). Racial breakdown was as follows: 58.3% White, 25.0% Black, 9.3% Multiple Races, 2.8% Asian, 0.9% American Indian or Alaskan Native, and 3.7% Other or unknown. 9.3% of the sample identified as Latina or Hispanic. (See Table 1).

### Total and Macronutrient Intake

Total energy intake: The first step of the model (base model) included covariates and the second step (full model) added the predictors of family adaptability and cohesion. Neither the base (*F*(2, 105) = 0.63, *p* = 0.534) nor full (*F*(4, 103) = 0.69, *p* = 0.598) models significantly predicted total energy intake. The full model accounted for only 2.6% of the variance in total energy intake. Adaptability (β = −0.04, *p* = 0.683) and cohesion (β = 0.13, *p* = 0.222) were not significant contributors to the full model.

Protein: Covariates (first step) explained 5.4% of the variance in percentage protein consumed but the base model was not significant overall, *F*(3, 104) = 1.98, *p* = 0.12. Adding adaptability and cohesion to the model improved model fit and accounted for an additional 6% of the variance in percentage of protein consumed (Δ*R*^2^ = 0.06, *p* = 0.038) and the full model was significant, *F*(5, 102) = 2.59, *p* = 0.030. Percentage of body fat was a significant predictor of percentage of protein consumed (β = 0.24, *p* = 0.016) as was family adaptability (β= 0.26, *p* = 0.015), but not family cohesion (β = −0.18, *p* = 0.081) (see Table 2). Specifically, lower percentage of body fat and worse family adaptability were both significantly related to less percentage of protein consumed.

Carbohydrates: The first step of the model explained 7.2% of the variance in percentage of carbohydrates consumed and the base model was significant, *F*(3, 104) = 2.70, *p* = 0.049. Adding adaptability and cohesion to the model improved model fit and accounted for an additional 5% of the variance in percentage of carbohydrates consumed (Δ*R*^2^ = 0.053, *p* = 0.049) and the full model was significant, *F*(5, 102) = 2.93, *p* = 0.016. Total energy consumed was a significant predictor of percentage of carbohydrates consumed (β = −0.24, *p* = 0.011) as was family adaptability (β = −0.26, *p* = 0.015), but not family cohesion (β = 0.12, *p* = 0.232). Specifically, less total energy consumed and worse family adaptability were related to higher percentage of carbohydrates consumed, even when accounting for all other variables in the model.

Fat: The first step of the model explained 10.8% of the variance in percentage of fat consumed and the base model was significant, *F*(3, 104) = 4.20, *p* = 0.008. Adding adaptability and cohesion to the model did not significantly improve model fit (Δ*R*^2^ = 0.031, *p* = 0.169). The full model was significant, *F*(5, 102) = 3.28, *p* = 0.009, but adaptability and cohesion were not significant predictors. Significance was driven by total energy consumed, (β= 0.32, *p* = 0.001), with more total energy consumed related to higher percentage of fat consumed (see Table 2).

Follow-up analyses: Follow-up analyses were conducted for girls with a BMI percentile indicating overweight status (above 85th percentile) and with girls reporting four or more LOC eating episodes in the past 28 days. Follow-up analyses were conducted to assess whether results in the sub-groups would differ from the full sample. A total of 101 girls in the study had a BMI percentile above the 85th percentile and 37 girls reported four or more LOC eating episodes. Neither those with overweight nor those reporting four or more LOC eating episodes differed from the entire sample for any outcome.

## 4. Discussion

In this analysis of perceived family functioning and objective energy intake, we found that adolescent girls with reported LOC eating who perceived their families as being less adaptable consumed a lower percentage of energy from protein and a higher percentage from carbohydrates. Perceived level of family cohesion did not significantly relate to test meal intake measures. We found that familial adaptability was associated with degree of carbohydrate and protein intake, but not with total energy or degree of fat intake. These results are generally consistent with data comparing youth with and without LOC eating, such that those with LOC eating consume more energy from carbohydrate and less from protein [11,50,51]. Given that all participants in our sample reported LOC eating, and that poorer adaptability was specifically linked to macronutrient intake that may promote excess weight [52,53,54,55], interpersonal functioning linked to less familial flexibility may provide a more fine-grained understanding of the interpersonal model. Indeed, adolescent girls with LOC eating [15,56] and adults with binge eating [57] often describe rigidity within families. Supporting this notion, low levels of familial adaptability have been shown to correspond with high levels of stress [58] and thus, it is possible that girls consume higher amounts of carbohydrate-rich “comfort” food to cope with the stress of a relatively inflexible family environment. Likewise, protein consumption has generally been associated with healthier body weight [59,60] and eating patterns [61,62,63] and may thus explain how poorer family adaptability was related to adolescents’ lower protein intake. Of note, the relationship between family functioning and energy intake accounted for only a small proportion of variance. This could be due to family functioning being a global construct that may be persistent whereas energy intake in the laboratory is situational (e.g., may be affected by hunger, current mood, environment, etc.). As a result, future research including a more naturalistic measure of eating behavior outside of the laboratory, such as ecological momentary assessment, is warranted.

The lack of an association between family cohesion and intake was unexpected. Previous studies have found that higher cohesion correlates with healthier food intake [30,31,32,64]. It is possible that the nature of our sample and test meal design may explain differences in results. Previous studies were primarily conducted in children and adolescents who were not selected for disordered eating behaviors. Moreover, studies based on self-reported intake were focused on eating in general, as opposed to a meal designed to capture a binge episode. It is possible that we would have found a significant link with family cohesion had we assessed girls’ intake during a “normal” test meal. Indeed, our prior research has demonstrated that girls with overweight and reported LOC eating can distinguish between “binge” and “normal” meals in the laboratory [11]. However, this notion requires testing. Furthermore, perceived cohesion has been linked to more meals eaten as a family [31] and thus, authors of prior studies suggest that the cohesion–healthy eating relationship might be interpreted within this family-eating context, which promotes healthy food consumption [65,66]. Therefore, another possible explanation for our findings is that given that youth with LOC eating tend to consume fewer regular meals compared to those without LOC eating [67], cohesion may be less relevant with regard to intake among adolescents who report LOC eating behaviors in the context of a meal modeled to capture a binge episode. Indeed, girls ate alone during the test meal. Further, data based on self-reports tends to find that binge episodes often take place when others are not present [39,68,69].

Strengths of this investigation include the objective measure of energy intake, use of DXA to assess body composition, and the diverse sample of participants of various ethnic groups and races. Limitations of the study include that the FACES is a self-report measure, which may be vulnerable to subjective bias. Also, the cohort contained only adolescent girls of above-average weight with LOC eating, potentially limiting the generalizability of the findings. As we did not include control samples of girls with or without overweight and without LOC eating, future research should address this gap. Lastly, given the cross-sectional nature of the results, no conclusions on temporality between perceived family functioning and intake can be made.

In conclusion, we found that adolescent girls with LOC who perceived their families as being relatively less adaptable consumed significantly more carbohydrates and less protein from a test meal that modeled a LOC episode. The findings of this study underscore the importance of addressing family functioning in the clinical setting and potentially including family members in interventions involving the modification of adolescent girls’ eating behaviors. Future prospective research is required to elucidate whether familial functioning plays a role in the development of adverse outcomes and intervention response.

## Figures and Tables

**Table 1:** Descriptive characteristics of participants.

	*N* = 108
Age, year *	14.5 (1.7)
BMIz *	1.54 (0.33)
**Race/Ethnicity, %**	
White	58.3
Black	25.0
Asian	2.8
American Indian	0.9
Hispanic	9.3
More than one race	9.3
Other/Unknown	3.7
FACES Score *	
Adaptability	31.6 (6.2)
Cohesion	26.2 (8.1)
**LOC Eating Episodes**	
Episodes 1 month pre-test meal *	4.69 (6.2)
Proportion of youth reporting at least 4 episodes per month in 3 months pre-test meal	14.8%
Test Meal *	
Total kcals consumed	1,177.3 (453.7)
Kcal from protein %	12.9 (3.2)
Kcal from carbohydrates %	50.9 (8.2)
Kcal from fat %	36.2 (7.0)
Pre-meal hunger (Scale 1–100)	62.7 (18.6)
Pre-meal depressive symptoms (Scale 1–5)	0.44 (1.02)
Post-meal subjective evaluation of LOC eating (Scale 1–5)	3.94 (1.1)

* = Mean (SD).

**Table 2:** Hierarchical regression for total intake and macronutrients.

Outcome Variables																	
		Total Intake				Protein				Carbohydrates				Fat			
Model	Predictor Variables	β	t	*p*	∆R^2^	β	t	*p*	∆R^2^	β	t	*p*	∆R^2^	β	t	*p*	∆R^2^
1	Constant		13.0	0.00 *	0.01		3.82	0.00 *	0.05		7.87	0.00 *	0.07		1.47	0.15	0.11 ^†^
	Total lean fat	0.06	0.61	0.54		−0.03	−0.31	0.76		−0.06	−0.62	0.54		0.08	0.82	0.42	
	Total % fat	−0.08	−0.79	0.43		0.21	2.18	0.03 *		−0.15	−1.55	0.12		0.07	0.74	0.46	
	Total intake (kcals)					−0.05	−0.53	0.60		−0.23	−2.45	0.02 *		0.32	3.39	0.00 *	
2	Constant		11.77	0.00 *	0.01		3.34	0.00 *	0.06 ^†^		8.32	0.00 *	0.05 ^†^		1.02	0.31	0.03
	Total lean fat	0.007	0.69	0.50		−0.07	−0.69	0.49		−0.03	−0.27	0.79		0.05	0.55	0.59	
	Total % fat	−0.08	−0.81	0.42		0.24	2.45	0.02 *		−0.17	−1.81	0.07		0.09	0.92	0.36	
	Total intake (kcals)					−0.03	−0.35	0.73		−0.24	−2.60	0.01 *		0.32	3.46	0.00 *	
	Adaptability	−0.04	−0.41	0.68		0.26	2.47	0.02 *		−0.26	−2.49	0.02 *		0.20	1.90	0.06	
	Cohesion	0.13	1.23	0.22		−0.18	−1.76	0.08		0.12	1.20	0.23		−0.07	−0.70	0.49	

† = Model significant; * = *p* < 0.05; Note: Percentages of protein, carbohydrates, and fat intake were arcsine-square root transformed for analyses.
